# The Role of Dog Population Management in Rabies Elimination—A Review of Current Approaches and Future Opportunities

**DOI:** 10.3389/fvets.2017.00109

**Published:** 2017-07-10

**Authors:** Louise H. Taylor, Ryan M. Wallace, Deepashree Balaram, Joann M. Lindenmayer, Douglas C. Eckery, Beryl Mutonono-Watkiss, Ellie Parravani, Louis H. Nel

**Affiliations:** ^1^Global Alliance for Rabies Control, Manhattan, KS, United States; ^2^Centers for Disease Control and Prevention, Atlanta, GA, United States; ^3^Humane Society International, Washington, DC, United States; ^4^National Wildlife Research Center, United States Department of Agriculture, Fort Collins, CO, United States; ^5^World Animal Protection, London, United Kingdom; ^6^Department of Microbiology and Plant Pathology, University of Pretoria, Pretoria, South Africa

**Keywords:** canine rabies, dog population management, dog population control, free-roaming dogs, stray dogs, responsible dog ownership, sterilization

## Abstract

Free-roaming dogs and rabies transmission are integrally linked across many low-income countries, and large unmanaged dog populations can be daunting to rabies control program planners. Dog population management (DPM) is a multifaceted concept that aims to improve the health and well-being of free-roaming dogs, reduce problems they may cause, and may also aim to reduce dog population size. In theory, DPM can facilitate more effective rabies control. Community engagement focused on promoting responsible dog ownership and better veterinary care could improve the health of individual animals and dog vaccination coverage, thus reducing rabies transmission. Humane DPM tools, such as sterilization, could theoretically reduce dog population turnover and size, allowing rabies vaccination coverage to be maintained more easily. However, it is important to understand local dog populations and community attitudes toward them in order to determine whether and how DPM might contribute to rabies control and which DPM tools would be most successful. In practice, there is very limited evidence of DPM tools achieving reductions in the size or turnover of dog populations in canine rabies-endemic areas. Different DPM tools are frequently used together and combined with rabies vaccinations, but full impact assessments of DPM programs are not usually available, and therefore, evaluation of tools is difficult. Surgical sterilization is the most frequently documented tool and has successfully reduced dog population size and turnover in a few low-income settings. However, DPM programs are mostly conducted in urban settings and are usually not government funded, raising concerns about their applicability in rural settings and sustainability over time. Technical demands, costs, and the time necessary to achieve population-level impacts are major barriers. Given their potential value, we urgently need more evidence of the effectiveness of DPM tools in the context of canine rabies control. Cheaper, less labor-intensive tools for dog sterilization will be extremely valuable in realizing the potential benefits of reduced population turnover and size. No one DPM tool will fit all situations, but if DPM objectives are achieved dog populations may be stabilized or even reduced, facilitating higher dog vaccination coverages that will benefit rabies elimination efforts.

## Introduction

Domestic dogs (*Canis lupus familiaris*) are responsible for over 99% of human deaths due to rabies (1). The key objective of a successful canine rabies elimination program is to maintain a high enough level of rabies vaccination coverage to interrupt rabies transmission within a defined dog population. This in turn reduces the incidence of rabies among human populations (1).

Stable dog populations with relatively low turnover rates make continuous vaccination coverage highly feasible. However, in many countries in which canine rabies persists, economic barriers and cultural attitudes toward dogs enable the maintenance of large free-roaming dog populations (2). Where the size of the free-roaming dog population is large and turnover is high, regularly vaccinating a large enough proportion of the population to achieve rabies elimination is a huge challenge. The stabilization of dog populations, and, in some cases, the humane reduction of the population over time to a manageable size, would be valuable adjuncts to long-term canine rabies control strategies.

Dog population management (DPM) is a multifaceted concept which aims to improve the health and well-being of free-roaming dogs, reduce problems they may cause, and may also set goals to reduce the size or turnover of the population (3). DPM may be enacted for numerous animal welfare, public health and safety, and economic reasons. These reasons include reducing the incidence of human bite injuries, secondary infections, and death; reducing or eliminating the transmission of rabies and other zoonotic diseases; reducing the level of noise and the amount of fecal contamination of the environment; reducing the incidence of traffic accidents; limiting the amount of negative publicity directed at governments; and minimizing the impact of reductions in tourism associated with free-roaming dog populations (2–5). Therefore, DPM programs can have one or more goals, depending upon specific situations, and these may or may not include permanently reducing the size of a dog population. Tools to achieve DPM objectives are humane and intended to produce a long-term positive impact on free-roaming dog populations, in contrast to dog culling (6).

Whether and how to manage dog populations effectively within rabies control programs has become the subject of debate (7, 8). However, because of the potential implications of DPM measures for the sustainability of rabies control programs, the World Organisation for Animal Health (OIE) recommends DPM as an integral part of such programs (9). Incorporating a DPM program with potential to improve animal, human, and environmental health into a rabies control program may increase motivation to tackle the issues and bring on board more stakeholders to support efforts.

Assuming that a rabies vaccination program is in place or being planned, this review aims to assess how different DPM tools might benefit rabies control programs and how to choose the most appropriate tools. We also consider available evidence for the impact of DPM measures on the health, stability, and size of dog populations. Finally, we review the feasibility and costs of implementing these interventions. This review does not aim to give prescriptive advice, but presents the available evidence, and allows program designers to assess, for their particular situations, whether it may be beneficial to integrate DPM into their rabies control planning.

## Domestic Dogs and Responsible Dog Ownership (RDO)

Domestic dog populations are dependent on people for food, either directly or indirectly (e.g., through open garbage dumps), and their presence and movements are linked tightly to human actions (10–12). Thus, dog population size is heavily dependent on human behavior, and dog-related problems are consequences of human behavior.

In most settings where this has been studied, the majority of dogs (even if free-roaming) have identifiable owners, which may be either individuals or community groups (12, 13 and see Section “Which DPM Approaches Might Be Suitable in a Particular Setting?”). RDO involves owners accepting their duties to provide the resources (e.g., food, water, shelter, health care, social interaction, exercise, and opportunity for natural behaviors) necessary for dogs to maintain an acceptable level of health and well-being in their environments; to act in accordance with the legislation in place (including vaccination); and to minimize any risks (aggression, disease transmission or injuries) that dogs may pose to communities, other animals, or the environment (3, 4). Dogs may have a single owner or be cared for collectively by a family or a group of individuals (3).

Widespread practice of RDO at a community level will be the most effective way to achieve DPM objectives, as long as veterinary services (such as vaccination and sterilization) are accessible and affordable to owners. Empowerment of communities with the knowledge to actively participate in DPM programs that are suited to the setting will be critical to ensuring DPM programs’ success and sustainability. However, the intended impacts of RDO may be severely compromised where access to veterinary services is poor and in settings where a large proportion of dogs do not have responsible owners. For unowned dogs and those without responsible owners, the responsibility for providing veterinary care often falls upon government entities and non-govermental organizations (NGOs).

## What are the Theoretical Benefits of DPM Programs for Rabies Control?

The primary focus of a rabies control program in dogs is vaccination (1). Mass dog vaccination programs generally aim for a 70% vaccination coverage so that between campaigns, levels of protection stay above the threshold necessary to prevent ongoing transmission (14, 15). High enough levels of canine vaccination will break the enzootic cycle of transmission between dogs, protecting them and their communities from rabies and leading to elimination of the disease. There is now much evidence that achieving 70% vaccination coverage, even where dog population turnover is high, is feasible (16–18), but it can be challenging.

Rapid population turnover (due to high death rates) of both owned and unowned dogs can present a significant challenge for the maintenance of high vaccination coverage (6, 17). Puppies comprise large proportions of dog populations in many rabies-endemic areas, even where almost all dogs are owned (12, 19, 20). A longitudinal study in West Bengal, India, found that 67% of new puppies died within 4 months and 82% within their first year (21). A survey in Nepal estimated 60% puppy mortality (13), and studies in Latin America and Africa have reported population-wide death rates as high as 30% per year (17, 20, 22, 23). All dogs, including puppies, can transmit rabies and should be vaccinated during mass vaccination campaigns. High population turnover means that vaccinated dogs often die and annual campaigns are generally required to vaccinate their replacements (24, 25).

There are several ways in which effective DPM programs could theoretically benefit rabies control activities.

### Maintaining Vaccination Coverage

There is unlikely to be a clear impact of reduced dog population density on rabies transmission rates between dogs [measured as R_o_ (26)]. However, DPM programs that reduce the dog population size will make reaching 70% vaccination coverage of dogs much easier and less costly. This is particularly true of free-roaming dogs that are difficult to handle or unowned dogs which are often the most time consuming to vaccinate. DPM that improves the health and longevity of vaccinated dogs will, by reducing the population death rate, also reduce population turnover and allow vaccination coverage to be maintained more easily, even if the population size remains unchanged (17, 18).

### Reducing Bite Incidents

In practice, in rabies-endemic areas any dog bite should be considered a possible exposure to rabies, and demand for human post exposure prophylaxis (PEP) is one of the major costs associated with canine rabies (27). Until canine rabies can be eliminated, DPM that reduces the incidence of dog-bite injuries will reduce the demand for PEP and, therefore, increase cost effectiveness of control programs.

Canine aggression that results in dog bites can have many different causes, including fear, resource guarding, pain, territorial behavior, maternal guarding of puppies, play aggression, and predatory behavior (28, 29), with fear the most common trigger of aggression (30). Some forms of aggression, such as inter-male aggression and female puppy-guarding aggression, are hormonally related and sterilization may reduce them (29, 31). However, the impact of different DPM methods on bite incidence may not be easily predicted. An analysis of free-roaming male dog behavior changes following castration in Chile showed no reduction in overall aggression as a result of surgical sterilization, and a significant increase in dog-to-dog aggression as a result of chemical castration (32).

Dog bites may be provoked by people, and high dog-bite incidences can feed a cycle of intolerance toward free-roaming dogs that makes the dogs more aggressive in return (2). Temporary marking of recently vaccinated dogs and permanent marking of sterilized animals can play a role in improving community acceptance of dogs and reducing cruelty toward them. Education and RDO programs aimed at changing community attitudes and behaviors toward dogs as part of a DPM program may result in reduced dog-bite incidence.

### Increasing Support for Interventions

A combined program of DPM and rabies control (for example, one that seeks to reduce nuisance dog behavior, dog-bite incidence, and rabies transmission), may have much broader appeal to the public and health authorities or other stakeholders than a single program. For this reason, introducing DPM measures that improve animal welfare into rabies control programs may bring on board additional partners with expertise and funding. Evidence of this is provided by animal welfare NGOs which implement rabies control programs using DPM measures as their main strategy, where there might otherwise be no program at all (33–36).

### Increasing Program Sustainability

Appropriate, acceptable DPM programs can allow communities to live in better balance with the free-roaming dogs in their environments. It is easier to maintain high vaccination coverage in populations of dogs that are healthier, live longer, and are more familiar with their environments (17, 18). Healthier, better managed dog populations may elicit more positive public attitudes toward those dogs (2), and increase the likelihood that communities seek rabies vaccinations for their dogs (17). Anecdotal reports from one community suggest that where DPM has achieved a reduction in dog population size, the remaining dogs are better cared for (37). Dogs that are well fed and cared for may in turn also mount a better immune response after vaccination (25).

## Mass Dog Culling is Not an Effective DPM Tool

Mass dog culling is still used as a misguided emergency response to rabies outbreaks, based on the mistaken belief that reducing the size of dog populations will reduce rabies transmission (38). In fact, mass dog culling has been shown to have no long-term impact on the control of rabies within cities (36, 39, 40) or across countries such as Ecuador, Indonesia and Bangladesh (19, 41–43). When modeled in realistic scenarios, culling is not as effective as sterilization programs at reducing population size in the long term (44). This is because culling does not address the source of new or replacement animals, and has only a temporary effect on population size. Furthermore, rapid dog replacement rates have been documented in some areas following culling, leading to a younger population of generally rabies-susceptible dogs (45, 46).

Indiscriminate culling of dogs in communities where rabies vaccination programs are operating is likely to remove vaccinated dogs from communities, resulting in lower vaccination coverage and a counter-productive increase in rabies transmission as populations recover (7). Culling often meets with public resistance both within the local area and outside, especially as the methods employed are often inhumane (47). The result can be withholding of dogs from rabies vaccination efforts during current and future campaigns. People may even move dogs away from culling zones, a measure which has been documented to spread rabies (15). Some methods of culling, such as poisoning, may pose threats to public health. Culling operations can also be expensive (19, 42, 48) and harmful to tourism (49). For these reasons, the indiscriminate culling of dogs is now universally condemned as a means to control rabies (1).

## What are the Benefits and Drawbacks of Humane DPM Tools?

The culling of dogs has now been replaced in some settings by a variety of humane DPM approaches that aim to exert sustainable, positive impacts on dog populations and the communities in which they live (6). DPM tools such as vaccination and other disease control methods, control of access to food (habitat control), the promotion of RDO, prevention and control of reproduction, identification and registration of individual dogs, the availability of shelters, rehoming centers and holding facilities, and the passage of legislation can interlink with each other to create effective DPM programs. Much of the motivation for DPM in rabies control efforts comes from the desire to reduce the size or the turnover of the free-roaming dog population to make effective vaccination more feasible. For this reason, reproductive control is usually a primary objective, but other efforts that increase longevity and reduce population turnover will also support rabies control efforts.

### Tools for Reproductive Control

Both permanent and temporary methods of reproductive control are available (summarized in Table 1). Permanent sterilization is preferable in most settings where rabies control is the objective, but temporary contraceptive methods will be more appropriate where owners may wish to breed dogs in the future (50).

**Table 1:** Reproductive Control tools currently available that can be incorporated into dog population management.

Reproduction control tool	Required resources	Targeted population	Product	Targeted sex	Duration of infertility	Potential negative consequences	Cost	Reference
Surgical sterilization	– Veterinary personnel – Aseptic techniques – Suitable operating and recovery facilities – Medications	Unowned or owned dogs, depending on program structure	NA	Male and female	Permanent	– Surgical complications – Post-surgical complications	$6–$100+ (see Table 4) Cost	(33, 40, 51–54)

Injectable contraceptives	– Veterinary or trained/certified personnel for delivery and monitoring – Commercial product – Accessible veterinary service in event of complications	Unowned or owned dogs, depending on program structure	Zinc gluconate *(Zeuterin*^TM^/*Esterilsol^TM^*/*Neutersol^TM^)*	Male	Permanent	– Abscess at injection site – Temporary swelling of testicles	$15	(50, 55)

			Progestins [melengestrol acetate (MGA)]	Female	6 months	– Need for regular monitoring – Uterine infections, cancer, endometrial disease, depression, death		(50)

			Calcium chloride	Male	Permanent	– Temporary swelling of testicles, scrotal abscesses and necrosis necessitating surgical intervention – Risks associated with inaccurate or non-sterile compounding – Still considered experimental	Pennies	(50, 56–58)

Implantable contraceptives	– Veterinary personnel for delivery and monitoring – Commercial product – Accessible veterinary service in event of complications	Unowned or owned dogs, depending on program structure	Progestins (MGA)	Female and male	Up to 2 years	Females: – Induces estrus – 4 to 6 weeks to take effect – Need for regular monitoring – Uterine infections, cancer, endometrial disease, depression, death	$25–$75	(50)

			GnRH agonists (*Suprelorin^TM^)*	Female and male	Up to 27 months	– Initially causes estrus and ovulation	$100	(50)

Oral contraceptives	– Responsible owner – Daily treatments – Accessible veterinary service in event of complications	Owned dogs	Megestrol acetate	Female	Daily	– Requires daily treatments at specific times of cycle – Need for regular monitoring – Uterine infections, cancers, and depression		(50)

Physical confinement	– Trained, responsible owner – Suitable place for dog confinement	Owned dogs	NA	Female and male	Not applicable	– If confinement fails, pregnancy may result – Welfare and safety concerns as females in season still attract males – Welfare concerns if not correctly confined	Free	

Surgical sterilization is currently the most widely used option. Surgical procedures to remove reproductive organs must be carried out by qualified veterinarians using good aseptic techniques and pain management throughout and after the procedures (3). In settings where the majority of dogs are family-owned, fixed point sterilization campaigns may have great success. In settings where there are large numbers of community-owned or unowned dogs, programs that capture, sterilize, vaccinate and return free-roaming dogs to their communities may be more effective. As dogs are territorial animals, it is assumed that returning sterilized dogs to their original locations helps to prevent new, fertile, and unvaccinated dogs from occupying these areas. Standard operating procedures generally recommend this practice (59). In some instances programs are referred to as “dog managed zones,” where the aim is to establish stable populations of sterilized, vaccinated dogs within defined areas (35). Whether territories are effectively guarded or not, this process means that more of the ecological niches available to dogs in a particular area will be occupied by sterilized, vaccinated dogs, reducing the proportion of niches available to young, unvaccinated dogs. Ecological models have demonstrated that this leads to a reduction in the number of young, unvaccinated dogs in those areas (44).

Surgical sterilization provides lifelong reproductive control and may also reduce problematic behaviors such as some forms of aggression or the propensity for specific dogs to roam (28, 31). It could improve animal welfare by reducing the dumping and killing of unwanted puppies and the stress experienced by female dogs that produce litters repeatedly. Surgical sterilization has been documented to reduce the lifelong probability of cancers and other diseases in both male and female dogs and can also increase life expectancy (6, 31). On the other hand, if there are not enough skilled veterinarians with access to recommended drugs and equipment, the procedures could fail to achieve sterilization and, combined with post-operative complications, could increase animal suffering.

Population simulation models predict that the effect of sterilizing females is far more significant than that of sterilizing males in terms of reducing population sizes (60, 61). Dog population sizes can be reduced where enough female dogs are sterilized, but this is a long-term goal for which very high throughput surgery is often required. It is important that if only females are targeted for sterilization, male dogs should still be vaccinated to prevent rabies.

A variety of non-surgical methods can be used to prevent reproduction. These include physical restraint of females and males, as well as injectable, implantable and oral contraceptives. The methods are summarized in Table 1 and their use for the management of free-roaming dogs is reviewed in more detail elsewhere (50, 62).

With the exception of physical restraint and dosing of oral contraceptives, all reproductive control methods should be implemented by trained individuals (e.g., veterinarians). Many of the newer tools are not widely licensed, experience and training in their use are limited and costs can be prohibitively high (50). Female dogs treated with hormone-based non-surgical methods must be monitored daily for evidence of pyometra (uterine infection) and other potentially life-threatening complications, and veterinary medical care must be accessible in the event that these occur (62). The administration of products with short-term contraceptive effects needs to be closely managed by responsible owners to be effective, and this method is not practical in most rabies-endemic countries. For unowned dogs, permanent sterilization will usually be required, and the costs and the feasibility of reaching enough dogs to achieve population-level effects must be carefully considered.

Surgical sterilization remains the most widely used technique as it produces a permanent solution and is available for both sexes. If population reduction or stabilization is the desired outcome, then high throughput sterilization focused on female dogs is necessary, together with some method of clearly identifying dogs that have already been sterilized. Sterilization of at least 70% of females is often mentioned as a target to achieve for population reduction, but this has no theoretical or practical basis. The coverage level necessary to achieve an impact on population size instead depends on the turnover characteristics of the local dog population. A study on the island of New Providence in the Bahamas estimated that for the population to reach equilibrium, 83% of females would need to be prevented from breeding (63).

The length of time required to achieve a desired outcome will also vary according to population turnover and sterilization efforts. Studies of sterilization programs in different settings have suggested that their full impact on reducing population size would not be achieved for over 30 years [for a shelter based spay/neuter campaign in the US (64)], up to 10 years [for sterilization of free-roaming dogs in Brazil (54)] and between 13 and 18 years [for sterilization of free-roaming dogs in India (34)]. Therefore, sterilization may be useful in reducing dog populations over a relatively long time period, but its impact will also need to be considered within the scope and timeframe of a rabies control program.

### Vaccination and Parasite Control

Reducing the incidence of canine diseases other than rabies such as canine distemper, and the prevalence of parasitic worms, may improve dog health and life expectancy and, therefore, reduce population turnover rates. Reducing the incidence of canine zoonoses also benefits public health. Many DPM programs routinely treat dogs with ivermectin to reduce parasitic infections and suffering due to itchy skin conditions (36, 51, 59, 65, 66). Anecdotal reports indicate that improving the body condition of dogs led to significant improvements in RDO and community acceptance of dogs in some settings (2).

### Controlling Access to Food

Based on the availability of resources (food, water, shelter) and human acceptance, there is an upper limit on the dog population size that can be supported by any environment (10). The dependence of the dog population on environmental resources such as waste food around markets and garbage dumps has been suggested to be high in some settings (19, 36, 67, 68) but very low in others (12, 69), depending on the quality of the waste food sources. There is some evidence that the percentage of ownerless dogs is higher around garbage dumps than elsewhere (10, 67). Free-roaming dogs may be frequently observed scavenging in waste, leading to claims that waste removal will help reduce the population (70, 71). However, without studies of the nutritional quality of waste food sources needed to sustain a population, it is unclear if these interventions will help. In Cameroon, residents associated open garbage dumps with an increase in stray dogs and, therefore, an increased risk in rabies transmission, although this was not confirmed empirically (68).

In one general dog population dynamics model, changing the parameter value of the upper limit of dog population size was identified as the most effective way to modify dog population dynamics of both owned and unowned dogs (72). While the owned dog population is unlikely to be reduced easily, reducing environmental food sources and shelters was expected to have a strong influence on reducing population size among ownerless dogs. However, if abandonment rates or other factors are not simultaneously changed, population size reduction will only be achieved by high death rates due to starvation (72).

Reducing access to food waste such as garbage in the streets, waste around abattoirs, butcher shops, and market areas, and protecting garbage dumps from scavengers have been suggested as practical, cheap, and sustainable ways to reduce free-roaming dog population sizes (73). There is a need to determine first whether food waste does in fact limit the size of a population, and any reduction of this food source must be gradual to avoid increased aggression between dogs over fewer resources, and to prevent starvation of existing animals or their migration to neighboring areas (3). This approach will also require public education (possibly supported by legislation) and may not work where dogs feed on other animals such as rats (74) or where dog populations are regularly fed by people. If free-roaming dogs are regularly fed by the community (75), changing attitudes and practices toward this activity may be extremely difficult, particularly in cultural settings such as Buddhist communities where feeding stray animals is perceived as a selfless act of kindness and generosity (76).

### Community Education, Engagement, and Empowerment

Dog ecology is integrally linked with human activities. The promotion of RDO coupled with the availability of vaccination and sterilization services could significantly reduce abandonment, the numbers of free-roaming dogs and the incidence of dog bites and zoonotic diseases (3, 4, 77). In the long term, RDO is key to the changes in human behavior that will allow DPM achievements to be sustained.

Where problems related to the dog population have been identified in or by a community, its involvement in developing a program and increasing access to information can help the community to identify the best options to deal with those issues. Supplying information about the benefits and practicalities of sterilization and vaccination, and how it will affect their dogs’ behavior, can help to change community attitudes, dispel myths that may be circulating and encourage owners to have their dogs sterilized and vaccinated. Awareness of solutions to dog-related problems may in itself empower communities to demand better access to veterinary services.

Community engagement initiatives are long-term investments, as the benefits of healthier and possibly smaller dog populations may not be seen for several years. Nevertheless, they still require resources. Educational materials need to be tailored to the community, taking into account cultural differences and literacy levels and utilizing appropriate networks for information dissemination. It takes time and resources to work out how to convey messages to different audiences, and the development of culturally appropriate materials across numerous languages can be a significant challenge. Helping communities to assume ownership of the DPM program enables them to become engaged and empowered. This maximizes the chances of creating and maintaining a successful, sustainable program.

Again, accessible and affordable veterinary services will be critical if programs are to achieve DPM goals.

### Identification and Registration

Registration and identification can be emphasized as part of RDO and are often linked to animal health programs such as mandatory rabies vaccination and traceability.

Registration of animals in a centralized database can be used to support the enforcement of legislation on vaccination, the reuniting of lost animals with owners, prevention of theft and illegal breeding and trade, and identification of owners of biting dogs (3, 4). The control of dog reproduction by sterilization can be encouraged through reduced registration fees for sterilized dogs.

In practice, dog registration systems require extensive and centralized data management systems and consistent input and maintenance if they are to be kept updated and effective. In settings with a high proportion of family-owned dogs this method may be effective even if many are free roaming, but unowned dogs and those more loosely owned by the community are very unlikely to be counted by registration programs. In most resource-poor settings and where turnover in the dog population is very high, registration systems may be impractical (20). Registration mandates may be viewed with great suspicion by the public and could be undermined. Thus, registration or identification strategies must be designed considering their context and implemented using good communication strategies and incentives to encourage participation and alleviate community mistrust. High registration fees may deter dog owners from complying with the scheme (78).

### Legislation

The creation and enforcement of RDO and dog breeding legislation can strongly support community-level efforts to tackle dog population-related problems (4). DPM legislation is a necessary element of the government’s engagement and is important for the effective management and sustainability of DPM programs. Legislation can be used to ensure DPM is carried out humanely, that culling is not used, that indiscriminate breeding and sale are prevented, that owners of biting dogs are held accountable, and that importation/exportation of dogs is controlled. Relevant laws may be divided across different statutes, laws or acts covering rabies or other diseases, dog ownership, stray animal management, waste management, and other features of DPM. Ideally, legal codes are designed with incentives for complying and punishments for non-compliance and are enforced by authorities working together with the program; fines levied are used to support the maintenance of the enforcement program.

However, legislative change can be a long and bureaucratic process. Enforcement of legal codes is frequently very challenging, especially where the personnel needed to enforce codes are in short supply. In addition, such mechanisms may fail if enforcement is not seen as a priority, corrupt officials are an issue, or the community members’ ability to pay fines is low.

### Shelters/Rehoming Centers

Many high-income country models of DPM rely on a model where free-roaming dogs are collected from the streets by authorities and taken to shelters or pounds, from where they are ideally collected by their owners or rehomed. Dogs whose owners no longer want them can also be surrendered to shelters. Both these methods reduce the free-roaming dog population. In shelters, there is the opportunity to sterilize and vaccinate animals before they are rehomed and to educate new owners in RDO.

In practice, however, the number of dogs admitted to shelters usually far outpaces the community’s capacity to rehome them (54). Shelters are expensive and time consuming to run, and once facilities are overwhelmed with animals, animal welfare standards can fall dramatically (3).

In areas where rehoming rates are low due to cultural practices or limitations in local resources, euthanasia in shelters will remain necessary in order to prevent animal welfare violations that are inherent to overcrowded, under-funded shelters. Even in high-income countries with well-established shelter adoption schemes the proportion of dogs euthanized can be significant. Limited data point to 10.4% of shelter dogs euthanized in the UK (79), over 30% in Australia (80), over 40% in Brazil (81), and 40–50% in the US (82).

The cost of running shelters can also be prohibitively expensive. The Humane Society of the US estimates that each year $2.5 billion is spent by humane organizations and $800 million to $1 billion is spent by animal control organizations on managing the pet overpopulation problem (82). An OIE survey of DPM strategies found that shelters were prohibitively costly for most low-income countries (38).

Finally, the availability of dog shelters that absorb unwanted dogs can counterproductively increase animal abandonment (3). This may be because people surrender dogs to the shelter, or instead abandon dogs to the street thinking that shelters will pick them up and take care of them. Shelters do not address the source of dogs, and dogs taken from the streets are quickly replaced by new puppies if enough breeding females remain or if dog abandonment rates are high.

Thus, for practical, economic, and welfare reasons, in most rabies-endemic settings alternatives to shelters must be explored fully prior to any commitment to build one (3, 54).

### Holding Facilities

Holding facilities aim to safely, but temporarily, house dogs that will generally be returned to owners or to the streets. Such facilities can be beneficial for safely assessing aggressive or sick animals, including those suspected of rabies which might otherwise transmit the disease. These types of facilities can also be centers for safe and humane euthanasia of animals that are a threat to people, or have no chance of healthy lives in their communities. They can also serve as centers where street dogs are sterilized and vaccinated before being returned to the streets.

### Euthanasia

Ideally, euthanasia should be reserved for animals who are incurably ill, or whose suffering due to behavioral problems or lack of guardianship cannot be alleviated with available resources. Unfortunately, many dogs are euthanized as a means of population control as well. When the decision for euthanasia is made, it must be carried out by qualified veterinary staff with access to the necessary drugs and training in humane handling and euthanasia. Robust euthanasia policies and legislation can prevent the indiscriminate culling of dogs by defining clearly the only circumstances when euthanasia is acceptable, and this can build public trust in DPM programs (3). However, euthanasia deals only with the symptoms and not the causes of dog population problems and will not solve the underlying causes of overpopulation. Euthanasia can also be distasteful and stressful to professional animal caretakers (83, 84) and this can be a strong driving force for more acceptable DPM tools to be used.

## Which DPM Approaches Might be Suitable in a Particular Setting?

There can be many different relationships between people and domestic dogs within a community. Dogs may be owned for a variety of reasons, such as for companionship, for guarding the home or livestock, for hunting, or as a source of food. These relationships may affect the degree to which they are cared for and whether veterinary services or reproductive control may be sought by the owner [reviewed in Ref. (2, 6, 47)]. Where community ownership of dogs occurs, there may be some joint acceptance of responsibility for feeding these animals, but frequently this does not extend to full RDO (2, 13, 76). Understanding the ownership patterns and roles of dogs in a community is integral to choosing an appropriate DPM tool that will be acceptable to the community, thereby ensuring that it is as effective as possible.

Terminology around dog populations is varied and often misused. Dogs may be referred to as owned, unowned, semi-owned, free-roaming, unwanted, pet, feral, stray, community, village or neighborhood dogs. Local terminology may also apply. These terms are often not informative for the purposes of planning an effective DPM program. The often-used term, “stray” dog, is not consistently defined, sometimes being used interchangeably with free-roaming [which can include unowned, free-roaming owned, and owned lost dogs (4)] and elsewhere referring specifically to dogs with no owners.

Only two characteristics of dog guardianship are highly relevant to disease control and DPM: “confinement status” and “ownership status” [(3) and **Box 1**], and these are not mutually exclusive. Unowned dogs are never confined, but a free-roaming dog may be owned, community owned, unowned, or feral. In many countries, dogs are allowed to roam freely, but many of these dogs have owners [(10, 12–13, 24, 85, 86) and reviewed in Ref. (6)].

Box 1 **Key characteristics of dog guardianship for DPM purposes.**

CONFINEMENT STATUS

- A *confined dog* remains under owner control at all times, often within a home or walled compound, and is walked on a leash or maintained under control when outside those confines.
- A *partially free-roaming dog* spends part of its time confined to a home or a walled property, but is also allowed to freely roam in the community.
- A *fully free-roaming dog* is never confined to a home or walled property.

OWNERSHIP STATUS

- A *family (or individual)-owned dog* is a dog that a family or individual states is their property or claims a right over.
- A *community-owned dog* is a dog that more than one individual or family state is their property or claim a right over.
- An *unowned dog* is not claimed by anyone in the community. It may be accepted, tolerated or despised by the community.

Community-owned and family-owned roaming dogs can enjoy high standards of welfare when their needs are fulfilled. However, regardless of ownership status, free-roaming dogs are at higher risk for contracting diseases, injuries such as those caused by road-traffic accidents or acts of cruelty, and culling by governments or local communities, compared to owned confined dogs. This can lead, in turn, to owners failing to invest in their care (17), creating a vicious cycle of neglect and poor health.

Dog populations can vary across countries (6, 70, 78) and at more local scales (85). Understanding the composition of the dog population (such as the numbers of owned and unowned dogs in each category of confinement) and identifying which of these categories are the causes of the dog-related problems, will help to decide which DPM approaches should be considered (Table 2). Characterizing a dog population with terms like “stray” is of little use. The source of those dogs must also be considered to enable the design of a DPM program that will address the problem in a sustainable manner. Ownerless puppies may be abandoned (by owners, breeders or pet shops) or be born on the streets, and each cause may require a different management strategy. Finally, potential strategies need to be assessed for a number of features, including their acceptability to the community, their potential impact, the accessibility of dogs, animal welfare considerations, veterinary infrastructure needs and cost implications (50).

**Table 2:** Different sub-populations of dogs and factors relevant to dog population management.

Ownership status	Confinement status	Dependency on humans	Acceptance by community	Risk for rabies transmission (if unvaccinated)	Target for population reduction	Target for responsible dog ownership programs	Target for central-point sterilization	Target for capture–sterilize–release programs
Family owned	Confined	Fully dependent	High	Low	No	Yes	Maybe	No
Family owned	Partially free roaming	Fully or Semi-dependent	High	Moderate	No	Yes	Maybe	Maybe^a^
Family owned	Free roaming	Semi-dependent	High	High	No	Yes	Maybe	Maybe^a^
Community owned	Free roaming	Semi-dependent	High	High	Maybe	Maybe	Maybe	Maybe^a^
No owner	Free roaming	Independent	Variable, but lower	High	Usually yes	No (unless abandonment rates are high)	No	Yes

*^a^The suitability of this program will depend on obtaining owner consent where needed*.

No one DPM strategy should be expected to solve all problems or fit all situations (78). Knowledge, Attitude and Practices surveys of the community can be particularly helpful in elucidating what would be the most acceptable and therefore successful DPM components to apply in a particular setting (23, 75, 87). For example, if the unowned dog population is sustained mostly by owners dumping unwanted puppies, then legislative and educational efforts to increase RDO and central-point sterilization programs may improve the health and longevity of family-owned dogs and reduce the number of unwanted litters. If breeders are dumping unwanted animals, then better regulation of such establishments will be needed. However, if the unowned dog population is sustained by puppies born on the streets, then sterilization and release programs may be considered. Where there are plentiful food resources on the streets, tackling this issue may need to be prioritized in order for other DPM tools to have their anticipated impact.

Finally, it is important to understand that DPM strategies will not have the desired impact without community buy in. The whole community may not have a uniform attitude toward dogs, which can cause tension (2). It is important to assess exactly what the views are within a local community toward potential interventions. If members of a community want to own more dogs, more (generally unvaccinated) dogs will likely be bred or imported, even if DPM programs are being implemented. Assessing the dog population and understanding community attitudes are integral to development of a successful DPM program.

## Do DPM Tools have a Measurable Impact in Canine Rabies-Endemic Countries?

Community surveys in rabies-endemic countries often identify the need for improved DPM to help reduce the risk of rabies (6, 9, 17, 77), and small- and large-scale DPM interventions on free-roaming dogs are carried out in many places. However, before adding DPM interventions to an existing rabies control program, there is a need for solid evidence that DPM tools can have the desired impact on reducing dog population size or turnover, which will benefit rabies control objectives.

Although the impact of DPM programs is often assumed and sometimes informally reported (39, 51), it is often not critically assessed and even more rarely published following peer-review. A review of the literature on DPM recently compiled by the International Fund for Animal Welfare found very little information on the effectiveness of specific approaches to DPM, and found that the most comprehensive programs were generally not making their outcome data available (88).

The use of mixed DPM interventions, though often advisable, makes it very difficult to determine which of the individual interventions is responsible for success. For example, the successful impact of sterilization and release programs on reductions in rabies cases (39) is most likely due to the impact of dog vaccination and community engagement, not sterilization. While the establishment of a shelter in Erzurum City in Turkey has been credited with a 30% reduction in the number of bites from rabies suspect dogs (89), this shelter was primarily sterilizing, vaccinating, and then releasing free-roaming dogs, and the impact on rabies could be due to the vaccination component. A pilot program using Esterilsol^TM^ on male dogs in Raipura Island, Bangladesh was found to be flawed as it also involved extensive use of culling (90). Reported benefits of adequate waste removal practices on free-roaming dog populations could instead be explained by the ongoing collection of free-roaming dogs from the streets in that particular setting (70).

Available data on the effectiveness of DPM programs are summarized below, but their interpretation is still fundamentally limited by the lack of control areas.

### Injectable Sterilants

The injectable sterilant Esterilsol^TM^ has been used successfully in small scale safety and immunogenicity trials for male dogs in Todo Santos, Guatemala (91), and in Chile (55). However, no attempt has been made to assess its effect on longevity, population turnover, or individual dog behavior and aggression. The sterilization of male dogs is not expected to produce a reduction in population size, which is much more critically impacted by reductions in the reproductive capacity of female dogs (60, 61).

### Removal of Waste Food Sources

Food waste in garbage has been suggested as an important factor in maintaining dog populations (10, 68, 92), and better waste management has been implemented as part of some documented DPM programs (39). However, there is a lack of evidence of the impact of removal of food sources in garbage dumps and marketplaces on dog population size or rabies control.

### Leashing and Confinement

There is some evidence that in low-income countries, leashing or confinement of dogs can be both effective at reducing contact between dogs and well-tolerated during rabies outbreak situations, but after an outbreak is over it is less likely to be tolerated, as communities prefer dogs to roam freely (19, 93). Thus the value of confinement as a means to reduce dog populations is unlikely to be high in most settings, and there can be welfare implications for dogs depending on the method and duration of confinement.

### Awareness and Legislation

The purpose of legislation and awareness measures is generally to support other DPM measures and their individual impact is hard to assess. However, without legal enforcement and the awareness needed to build community participation, large-scale sustainable DPM programs will be very challenging. Poor results from DPM programs have been suggested to be the result of a lack of public awareness about the program (94). Public awareness and enforcement of dog ownership laws in the Philippines helped to increase the proportion of households that registered their dogs and stopped them from roaming freely. Concurrently, the demand for sterilization services from the community increased (95).

Among high-income OIE member countries surveyed, enforcement of dog registration laws was the chief tool used to support DPM tools, but use of laws was much less common in low-income countries (38). Most countries have legislation related to stray dog control, but there is huge variation, often incompliant with OIE animal welfare guidance and generally inadequately enforced [summarized in Ref. (96)]. The fact that legislation frequently still permits culling in the event of rabies outbreaks may well contribute to the lack of application of more effective means of DPM and rabies control. In the OIE member country survey mentioned above, 46 out of 76 countries stated that it is official policy to kill free-roaming dogs (38).

One notable example of comprehensive humane legislation on DPM is India’s Animal Birth Control (Dogs) Rules, which became law in 2001 (97). These laws stipulate that owners are required to control the breeding of their dogs, while municipalities and local authorities are required to sterilize and vaccinate street dogs, with the participation of animal welfare organizations, private individuals and the local authority. Appeals to local authorities relying on this legislation have been responsible for the proliferation of DPM programs in Indian cities (39).

### Surgical Sterilization

Most of the available data on DPM programs aimed at benefiting rabies control come from sterilization, vaccination and release programs, and there is evidence of some success (Table 3). However, these campaigns frequently do not report an impact on population size or dog characteristics such as longevity which could impact population turnover (Table 3). There is limited evidence of population size reduction, primarily from India (33, 34), but effects have not always been achieved (94) or maintained (98). Sterilization rates need to be maintained for many years to reach their maximum impact (34). Very few programs have reached out beyond cities, and very few have sustainable government support for their implementation.

**Table 3:** Available information on impacts of surgical sterilization programs on dog population characteristics.

Location and assessment dates	Coverage achieved	Reported impacts	Reference
**Not peer reviewed**			

Bali, Indonesia, 1998–2005	51%	None	(6)
Bangkok, Thailand, 2002–2005	Less than 30%	None	(99)
Sri Lanka, 2005	70–90%	None	(6)
Rosebud Reservation, USA, 2003–2010	Not measured	(Unmeasured) reduction in population size, 50% reduction in bite incidents, 75% reduction in complaints of cruelty to dogs, and increased demand for veterinary services	(51)
Kathmandu, Nepal, 2006–2012	47% of females	Overall population size reduction from 2006–2010 but no further impact to 2012, within zones mixed results found	(98)

**Peer reviewed**			

Gelephu and Phuentsholing towns, South Bhutan, 2012	56–58%	Majority of free-roaming dogs had healthy body and skin conditions	(100)
Dhaka, Bangladesh, 2012–2013	19.2–79.3% across 29 of 92 city wards	Neutered dogs tended to be healthier than intact dogs	(36)
Bangalore, India, 2000–2001	10.4%	None	(94)
Colombo, Sri Lanka, 2007–2010	Not measured	% Lactating females reduced from 8 to 1.1%. Slight increase in population size (possibly a rebound effect from ceasing of culling). Dog bites dropped by 33%, public perceptions of free-roaming dogs improved	(35, 40)
Pink city area, Jaipur, India, 1994–2002	65% of females	28% reduction in population size	(33)
Pink city area, Jaipur, India, 2003–2011	70–80% of females	Around 50% reduction in dog bites, associated with reduction in breeding females	(101)
Jodhpur, India, 2005–2007	61.8–86.5% across 6 areas	Dog population declines of 51%*, 40%, 39%*, 28%*, 3% (*significant)	(34)
Jodhpur, India, 2006	Not measured	Sterilized dogs had higher body condition scores, but worse skin conditions	(65)

## Cost Considerations

The primary tool of rabies control remains canine vaccination. While DPM can in theory benefit vaccination efforts, it also incurs considerable additional costs and requires additional technical skills. DPM programs require long-term commitment, and implementing two project aims can be logistically difficult. When limited budgets and personnel are stretched too far there is a risk that trying to tackle more than one goal detracts from the achievement of either. If expensive and time consuming DPM approaches detract from vaccination goals, or draw funding away from vaccinating a sufficient proportion of dogs, then rabies control efforts will be hindered. However, if overlapping interests draw in additional partners (such as animal welfare NGOs) or additional budgets (perhaps from different government sectors such as public safety) to strategically integrate DPM tools into a rabies control program, then this could be a very positive outcome.

Data on programmatic field costs of many DPM tools are uncommon, but some estimates of DPM by sterilization (which may include rabies vaccination even if not specified), are shown in Table 4. Although these costs of sterilization may not seem very large for an individual dog, given the scale necessary, full program costs can be high. For the four years of an intervention in Colombo City, Sri Lanka, costs within the animal sector were over $1 million, compared to $190,875 for the four preceding years (35).

**Table 4:** Published data on sterilization costs for high throughput programs.

Intervention	Location	Reported cost/dog	US$ cost/dog	Reference
Surgical sterilization + vaccination	Tamil Nadu, India	Rs. 1,164	$22	(53)
Surgical sterilization + vaccination	Jaipur, India	GBP 4.80	$8.83^a^	(33)
Surgical sterilization	Bhutan	Nu 288	$6.36	(52)
Surgical sterilization	Campinas, Brazil	Real 105	$33.34^b^	(54)
Surgical sterilization	Indian reservation, USA		$23–28	(51)
Surgical sterilization (including staff and infrastructure)	Several WSPA sites		$10.30–$52.00 (average $25)	(40)
Surgical sterilization	Costa Rica		$8–$12	(62)
	India		$15–$20	
	Quezon City, Philippines	P 1,000–1,500	$24–$36	
	Phuket, Thailand		$30	
	Palawan, Philippines		$11.02 (excl. boarding)	
	Bangkok, Thailand		$23.25	
	Beijing, China		$43.69–$203.89	
	Chennai, India		$14.11	
	Shanghai, China	800–1,000 yuan	$128–$160	
	Shanghai, China	800–1,200 yuan	$128–$192	
Pinhole castration	Uganda		$2.12	(102)

*Costs in US$ are as reported in the sources, except ^a^1GBP = US$ 1.84 (average for 2006); ^b^1 Real = US$ 0.30 (average for 2015); exchange rates from http://www.x-rates.com*.

Higher throughput programs can reduce costs per dog (33) (Table 4) but overall, there are insufficient data available on costs in different settings. Sterilization and release programs are usually focused on urban areas, where dog and human population densities likely make economies of scale more feasible and travel costs more reasonable. A rabies control intervention that involved sterilization as well as vaccination in selected cities in Tamil Nadu, India was not considered economically viable at the scale of the entire state (53).

Programs targeting only female dogs for sterilization (with vaccination of both sexes) will be a much more cost-effective way to reduce population size and turnover (44, 59–61) although this is uncommon in the studies listed in Table 3. In areas where the community keeps more male than female dogs (34, 61) this strategy will be even more effective at impacting population-wide demographics.

The source of funding will also need to be considered as well as the cost of interventions, in planning DPM interventions. As some canine rabies-endemic countries are considered to be middle income countries, there may be at least a proportion of dog owners who can pay for sterilization of their dogs through private veterinary services. Increased training of private and non-profit veterinarians in high throughput sterilization coupled with community engagement on RDO could benefit the wider goals of DPM by increasing access to these services. However, in the poorest countries, even a very low cost of sterilization is likely to be beyond the means of dog owners. In these settings governments and non-governmental organizations will need to fund any services to owned as well as unowned dogs. In many settings, the provision of free sterilization services could be used as a way to establish a model for more RDO, and once their value is established, owners could perhaps be asked to pay some contribution toward costs.

The scarcity of data on the costs of different DPM strategies and of their effectiveness in canine rabies-endemic settings severely limits assessment of their cost effectiveness (78), and where different tools are combined in a program the cost effectiveness of different components becomes even harder to disentangle.

Given the current high costs of sterilizing sufficient numbers of dogs to impact population turnover and size, it is likely that for most settings, sterilization is not a cost-effective additional technique to support a rabies control program. An exploratory model for rabies control in India concluded that canine vaccination alone was more cost effective than combined vaccination and sterilization (61). However, further exploration of the additional costs and indirect benefits of sterilization, improvements in waste management, treatment for skin and parasitic conditions, educational interventions and legislative interventions to support rabies control would be very valuable.

## DPM and Rabies Control Now and in the Future

Humane DPM tools offer the theoretical possibility of better integration of dogs into communities and a stabilization, or even reduction in size of dog populations where it is easier to maintain vaccination coverage.

Unfortunately, the main DPM methods successfully employed in most high-income countries (well-enforced breeding and RDO laws, encouragement of sterilization and removal of free-roaming dogs from the streets into shelters, supported by dog identification and registration) do not transfer easily to low-income settings (19, 38, 77). Laws may not exist, are not enforced, or have meager consequences; sterilization services are not always readily available or affordable; shelters quickly get overwhelmed where rates of adoption are low; and high turnover makes registration impractical. While the tools and lessons developed for rabies control in high-income countries may provide some insight, more cost effective and culturally appropriate methods must be considered for rabies control in low-income countries.

Where population reduction of free-roaming dogs is wanted by owners and communities, veterinary services are abundant, and political will and funding are sufficient to address the issue, there is evidence that high throughput sterilization and release programs can achieve population reduction (33, 34). However, where sterilization, vaccination and release programs do not reach 70% of dogs, additional vaccination must be encouraged to ensure that vaccination levels are sufficient to halt rabies transmission as quickly as possible (36, 94, 100). Combined sterilization and vaccination programs that are enacted as a rabies control strategy but fail to reach sufficient dogs will be very ineffective at achieving goals of reducing rabies transmission (94).

Where veterinary services and funding to pay for DPM programs are insufficient, theoretical arguments would suggest that waste management programs to reduce food resources for free-roaming dogs should be encouraged. Along with promotion of RDO to reduce free-roaming dog population sizes, waste management could be the best option to reduce dog populations and the spread of diseases in resource limited settings (3), but evidence of this method’s effectiveness is currently lacking.

It is possible that large-scale DPM success in most low-income countries will require the development of a cost-effective (non-surgical) safe and permanent sterilizing agent for female dogs. Such research is being actively pursued and progress is being made (50, 62, 103).

Currently, the most promising option for permanent sterilization of female free-roaming dogs is GonaCon, a single-dose GnRH-based vaccine, but issues over side effects require further work on its formulation (50). Small scale safety trials of GonaCon given along with rabies vaccinations have been completed in female dogs in Mexico (104) and on an American Indian reservation in the US (105), but there are as yet no data on its effects on fertility.

The availability of a safe and effective single-dose injectable sterilant for both sexes would enable provision of reproductive control as an additional service to owners during mass dog vaccination campaigns. Such a sterilant could also be delivered to ownerless dogs under a capture, sterilize, vaccinate and release model that did not require transportation to surgical centers. Such a tool could revolutionize DPM programs and, in some settings, rabies control as well. However, until such a permanent sterilizing agent becomes available, a safe and effective sterilant that lasted even 2–4 years could still be very beneficial to animal welfare and rabies control.

## Conclusion

Integrating DPM programs into rabies elimination programs could supplement the goal of breaking the rabies transmission cycle with the goal of stabilizing dog populations. In theory this is the most sustainable way to eliminate canine rabies, but three factors critically limit its wider implementation in practice. First, the clear lack of systematic data collection and the paucity of DPM program evaluation need to be addressed. Organizations currently conducting DPM programs in rabies-endemic countries should strive to improve their methods of evaluating impact (78) using available guidelines (106) and publish their findings in peer-reviewed journals. Second, there needs to be an improved understanding of the costs of current DPM tools and their benefits to rabies control in order that full cost effectiveness analyses can be conducted. Third, a single-dose, permanent, non-surgical sterilant that is safe and effective in female dogs would dramatically increase the possibilities for DPM to cost-effectively improve rabies control and elimination efforts. Armed with this knowledge, integrating DPM into rabies control programs in low-income countries could move the world closer to freedom from canine-mediated human rabies deaths.

## Author Contributions

LT drafted the initial manuscript. All authors revised it critically for important intellectual content and approved the version to be published.

## Conflict of Interest Statement

The authors declare that the research was conducted in the absence of any commercial or financial relationships that could be construed as a potential conflict of interest.
